# Transcriptome Analysis of Developing Wheat Grains at Rapid Expanding Phase Reveals Dynamic Gene Expression Patterns

**DOI:** 10.3390/biology11020281

**Published:** 2022-02-10

**Authors:** Jiantao Guan, Zhenyu Wang, Shaoshuai Liu, Xingchen Kong, Fang Wang, Guoliang Sun, Shuaifeng Geng, Long Mao, Peng Zhou, Aili Li

**Affiliations:** 1 National Key Facility for Crop Gene Resources and Genetic Improvement, Institute of Crop Sciences, Chinese Academy of Agricultural Sciences, Beijing 100081, China; xidagjt@126.com (J.G.); wangzhenyu89@126.com (Z.W.); liushaoshuai@caas.cn (S.L.); kongxingchen1990@126.com (X.K.); wangfang1213@126.com (F.W.); spy5920@126.com (G.S.); gengshuaifeng@caas.cn (S.G.); 2 Sino-Agro Research Station for Salt Tolerant Crops, Yellow River Delta, Kenli District, Dongying 257500, China

**Keywords:** wheat, grain expanding phase, transcriptome dynamics, homoeolog expression bias, time-series RNA-seq

## Abstract

**Simple Summary:**

Understanding the regulatory mechanism underlying grain development is essential for wheat improvement. The early grain expanding phase boasts critical biological events like embryogenesis and initiation of grain filling. RNA sequencing analysis of this developmental stage revealed dynamic expressions of genes related to cell division, starch biosynthesis, and hormone biosynthesis. An unbalanced expression among triads may play critical roles as shown by multiple enriched metabolic pathways. Our work demonstrated complex regulation mechanisms in early grain development and provided useful information for future wheat improvement.

**Abstract:**

Grain development, as a vital process in the crop’s life cycle, is crucial for determining crop quality and yield. The wheat grain expanding phase is the early process involving the rapid morphological changes and initiation of grain filling. However, little is known about the molecular basis of grain development at this stage. Here, we provide a time-series transcriptome profile of developing wheat grain at 0, 2, 4, 6, 8, and 10 days after pollination of the wheat landrace Chinese Spring. A total of 26,892 differentially expressed genes, including 1468 transcription factors, were found between adjacent time points. Co-expression cluster analysis and Gene Ontology enrichment revealed dynamic expressions of cell division and starch biosynthesis related structural genes and transcription factors. Moreover, diverse, differential and drastically varied expression trends of the key genes related to hormone metabolism were identified. Furthermore, ~30% of triads showed unbalanced expression patterns enriching for genes in multiple pivotal metabolic pathways. Hormone metabolism related genes, such as *YUC10* (YUCCA flavin-containing monooxygenase 10), *AOS2* (allene oxide synthase 2), *CYP90D2* (cytochrome P450 90D2), and *CKX1* (cytokinin dehydrogenase 1), were dominantly contributed by A or D homoeologs of the triads. Our study provided a systematic picture of transcriptional regulation of wheat grains at the early grain expanding phase which should deepen our understanding of wheat grain development and help in wheat yield improvement.

## 1. Introduction

Wheat (*Triticum aestivum*) is one of the most important crops worldwide and wheat grain provides approximately 20% of calories consumed by humans [1]. Along with the rapidly increasing population, the demand to improve wheat yield and quality is becoming more and more urgent. Wheat yield and quality are largely determined at early grain development, especially the first 14 days after pollination (DAP) because this period is critical for final wheat yield and quality and is featured by rapid grain expansion where the morphogenesis is largely established via continuous cell division and expansion and the endosperm starts to fill with starch [2,3,4]. Other important events like embryogenesis, the formation of syncytium, cellularization of the endosperm and the early grain-filling (i.e., starch and gluten accumulation) in the endosperm cells also occur at this time period [4]. Thus, the proper setup of the grain structure determines the subsequent grain fill and maturation in the next 45 DAP. Transcriptome study of the grain developmental process has been studied [1,5,6]. Yet, the transcriptomic dynamics of the grain enlargement phase are not well deciphered and a broader view of the molecular basis underlying the cell division and grain-filling initiation should contribute to wheat improvement.

Hormones, such as auxin (IAA), cytokinin (CK), brassinosteroid (BR), abscisic acid (ABA), and gibberellic acid (GA), have been found to be key regulators of grain development, especially the grain expanding phase, as has been widely demonstrated in Arabidopsis, maize, and rice [7,8]. For instance, elevated CK levels were found in endosperm cell divisions in the early stage of rice and maize grain development [9,10]. Auxin, for instance, exerts a central role in grain development due to its multiple effects on pattern formation, cell division and expansion [7]. Relocation of PIN1 and PIN7, two important auxin efflux transporters, has been shown to be functionally essential for embryo polarity establishment [11]. Moreover, BRand auxin are crucial for endosperm development, and many genes regulating grain size and endosperm development are correlated with levels and signaling of BR and auxin [12], while ABA was suggested to negatively mediate seed size by influencing the timing of endosperm cellularization [13]. However, few studies systematically investigated genes affecting hormone metabolism on the transcription level during the wheat grain enlargement phase.

Common wheat is a hexaploid with three sub-genomes A, B, and D [14]. The availability of high-quality genome sequences recently allowed detailed study of gene expression patterns in the polyploid wheat. Polyploidization often results in homoeolog expression bias that, in many cases, were caused by epigenetic modifications including DNA methylation and histone modifications [15,16]. A significant portion of triads (1:1:1 homoeologs from three subgenomes) exhibit homoeolog expression bias [15]. These unbalanced expressions of homoeologs often affect plant growth, development, and stress responses in wheat [16,17,18]. Despite this, much remains unknown about their potential biological contributions to grains at early development.

Here, we performed a study of RNA-seq analysis at six consecutive time points (0, 2, 4, 6, 8, and 10 DAP) that concur with the grain expanding phase. Co-expression cluster analysis of differentially expressed genes identified characteristic expression patterns of structural genes and transcription factors that were known in model plants to be involved in cell division and starch biosynthesis. Expression of hormone related genes exhibited synergistic functions of these important factors during wheat grain development. Moreover, homoeologs from the three sub-genomes were found to have unbalanced expression patterns participating in various important metabolic pathways. Therefore, our work provided new insights into the dynamic transcriptional regulation during the grain expanding phase in wheat and a useful resource for future gene function characterization and application in wheat yield improvement.

## 2. Materials and Methods

### 2.1. Plant Materials and Growth Conditions

Chinese Spring (CS) was used in this study and grown in the Institute of Crop Sciences (ICS) experimental station in Beijing (116° E, 40° N). Grains were collected at 0, 2, 4, 6, 8, and 10 days after pollinations (DAP) with three biological replicates. After briefly being frozen in liquid nitrogen, grains were stored in a −70 °C freezer until use.

### 2.2. RNA Extraction and Sequencing

Total RNA was extracted with TRIzol reagent (Invitrogen, Burlington, ON, Canada). Strand-specific RNA-seq libraries were constructed using TruSeq Stranded mRNA Library Prep Kit (Illumina, San Diego, CA, USA) to generate Illumina sequencing libraries according to the manufacturer’s instructions. Paired-end sequencing was performed on a HiSeq X Ten sequencer.

### 2.3. Identification of Differentially Expressed Genes

Raw reads were filtered using the fastp software with default parameters [19]. We aligned the clean reads to the reference genome of *Triticum aestivum* cv. Chinese Spring (IWGSC RefSeq v2.1. https://urgi.versailles.inra.fr/download/iwgsc/IWGSC_RefSeq_Assemblies/v2.1/ (accessed on 2 September 2021)) using the STAR program [20] and unique alignments with less than two mismatches were retained for transcript assembly by the Cufflinks software (v2.2.1. http://cole-trapnell-lab.github.io/cufflinks/releases/v2.2.1/ (accessed on 8 May 2021)) [21]. The assembled transcripts of all the samples were merged by TACO [22]. The number of reads mapped in each gene model were counted using the htseq-count script in HTSeq [23] based on the merged transcript. Gene expression levels were normalized as fragments per kilobase of transcript per million mapped reads (FPKM).

Differentially expressed genes (DEGs) between adjacent time points were identified using the R DESeq2 package [24]. Genes with adjusted *p*-value < 0.05 and an absolute value of log2 fold change ≥ 1 were considered as DEGs. For each comparison, the consecutively earlier time point was used as a control. Gene Ontology (GO) enrichment analysis was conducted using the R clusterProfiler package [25].

### 2.4. k-Means Cluster Analysis

We performed *k*-means cluster analysis by using Pearson’s correlation distance based on the scaled FPKM values of DEGs. Different values for *k* were validated and *k* = 10 was selected as the most optimal cluster number according to the highest overall Silhouette coefficient (Figure S1).

### 2.5. Genome-Wide Identification of Transcription Factor Family

Transcription factors (TFs) were predicted and clustered into 51 families using the PlantTFDB database [26] based on their protein sequences. The over-representation of a TF family was significant when the *p*-value of Fisher’s exact test was <0.01.

### 2.6. Comparison of Relative Expression Levels among the Three Homoeologs of Triads

A total of 14,284 triads were found to have homoeologs in all three subgenomes using OrthoFinder software [27] based on protein sequences (Table S1). We defined a triad as expressed when the summed FPKM value of the A, B, and D homoeologs was >1. According to the previously reported method [15], expressed triads at each time point were further classified into seven categories including (1) Balance: similar expressions across the triad; (2) A dominant, (3) B dominant, and (4) D dominant: expression of one homoeolog far beyond the remaining two, (5) A suppressed, (6) B suppressed, and (7) D suppressed: expression of one homoeolog far below the remaining two. Pathway enrichment analysis was performed based on the MapMan annotation using the R clusterProfiler package [25].

## 3. Results

### 3.1. Transcriptome Analysis of Wheat Grains at Expanding Phase

**Figure 1:**
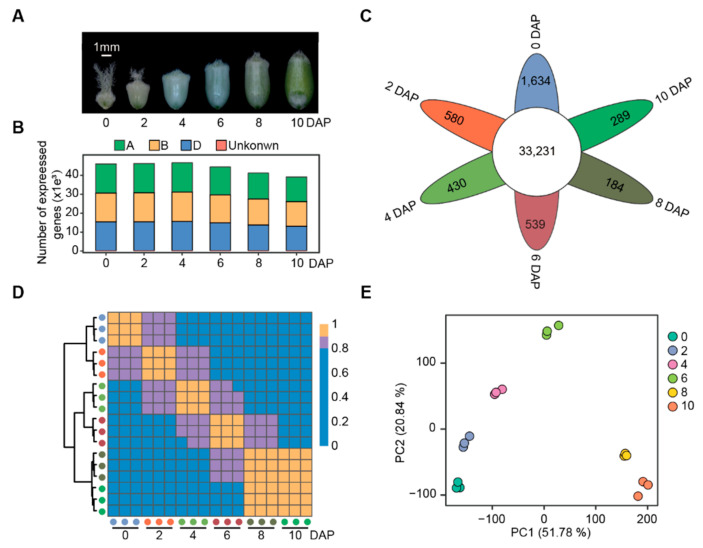
Overview of gene expression profiles during the wheat grain expanding phase. (**A**) Morphology of developing grains of *T.* *aestivum* cv. Chinese Spring at 0, 2, 4, 6, 8, and 10 DAP. (**B**) The number of expressed genes (FPKM > 1) in the A, B, and D subgenomes at each time point. (**C**) A Venn diagram showing the number of expressed genes shared among or specific to different time points. (**D**) Cluster dendrogram and Spearman correlation coefficient (SCC) of gene expression profiles among three biological replicates and different time points. The solid circle represents one biological replicate. The scale bar is the SCC. (**E**) Principal component analysis (PCA) plot of samples with three biological replicates at the six time points on basis of gene expression profiles.

To investigate the transcriptome dynamics during the grain expanding phase in common wheat, we performed RNA-seq assays at six time points when grains were evidently expanding. This process occurred during 0 to 10 DAP (days after pollinations) for Chinese Spring (CS) (Figure 1A). A total of 962.89 million high-quality (Q30 > 90%) reads were obtained, with an average of 53.49 million reads (>8 Gb) for biological replicates. These reads were aligned to the reference genome of the CS (IWGSC RefSeq v2.1), with the average rate of 90.24% unique alignment (Table S2). The uniquely mapped reads for each sample were used to determine the normalized expression level as fragments per kilobase of transcript length per million mapped reads (FPKM) of each protein-coding gene. Spearman correlation coefficient (SCC) between the biological replicates of different tissues varied from 0.95 to 0.99, reflecting the high-quality of these biological replicates (Table S3; Figure 1D).

A total of 54,182 expressed genes (FPKM > 1) were found in the grain expanding phase of CS, covering 50.68% of total annotated genes. Similar to previous studies [15], the number of expressed genes were similar among the three subgenomes (A: 18,086 genes, 51.00%; B: 17,963 genes, 50.18%; D: 17,925 genes, 52.31%). In addition, the number of expressed genes retained high levels in 0–6 DAP and then reduced starting at 8 DAP (Figure 1B). Of these expressed genes, 33,231 (61.33%) produced mRNAs detected in all the samples (Figure 1C), whereas 20,951 genes (38.67%) were expressed only at a specific time point, from 1636 at 0 DAP to 184 at 8 DAP (Figure 1C). Our data indicated that the early developing grains of wheat exhibited rapidly altering expression profiles, highlighting a reduction in gene activities in more mature grains.

To investigate the global differences in the transcriptome dynamics during grain expanding phase, we performed unsupervised hierarchical clustering analysis (Figure 1D) and principal component analysis (Figure 1E) based on the FPKM values of three biological replicates at the six time points. The time points showing a higher expression correlation are more similar in transcriptomes and biological processes. These two independent analyses gave conserved patterns, i.e., transcriptomes of adjacent time points were clustered together, particularly for samples at 8 and 10 DAP.

### 3.2. Expression Clusters and Cellular Function of Differentially Expressed Genes

To capture temporal changes during the wheat grain expanding phase, we compared gene expression levels among samples of the six time points. Using an adjusted *p*-value < 0.05 and an absolute value of log2 fold change ≥1 as thresholds, a total of 26,892 genes (covering 49.63% of the total expressed genes) were identified to be significantly differentially expressed genes (DEGs) between adjacent time points, with values ranging from 1436 (10 DAP vs. 8 DAP) to 12,521 (6 DAP vs. 4 DAP) (Figure S2; Table S4). Among identified DEGs, 9051, 8922, and 8783 genes (50.04%, 49.67%, and 49.00% of total expressed genes) came from the A, B, and D genomes, respectively. Of these DEGs in A, B, and D genomes, we found 5475, 5385, and 5421 homoeologs, respectively. In addition, DEGs were mainly distributed on chromosome arm ends (Figure S3). Only 58 genes were differentially expressed at all the time points, indicating complex transcriptional regulation of distinct processes during the grain developing period. Next, we conducted Gene Ontology (GO) enrichment for the DEGs between adjacent time points (Table S5). GO terms related to DNA replication (GO:0006260), cell wall biogenesis (GO:0042546), and cell growth (GO:0016049) were enriched in DEGs between 2 DAP vs. 0 DAP, 4 DAP vs. 2 DAP, and 6 DAP vs. 4 DAP. Moreover, DEGs related to nutrient reservoir activity (GO:0045735) and embryo development (GO:0009790) were found between 8 DAP vs. 6 DAP and 10 DAP vs. 8 DAP.

**Figure 2:**
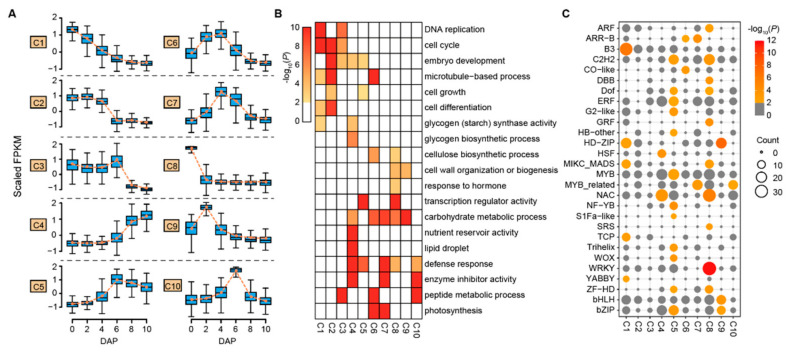
Co-expression clusters and over-representation of transcription factor (TF) genes in each cluster. (**A**) Boxplots showing the expression trends of genes in ten co-expression clusters (C1–C10) during the six time points. The orange dash line represents the average expression level of genes in each cluster. For box plots, the central line represents median values; bounds of the box are the 25th and 75th percentiles; whiskers were 1.5 * IQR (IQR: the interquartile range between the 25th and 75th percentile). (**B**) Heatmap illustrating the enriched GO terms of genes in ten clusters. The GO term with *p*-value < 0.01 (Fisher’s exact test) was considered to be significant. Cells of white-color in the heat map represent insignificant GO terms. (**C**) Number and over-representation of TF genes of 28 TF families in each of the ten clusters. Circle size is correlated with the number of TF genes in 28 TF families. Circle color represents the −log10(*p*) of the enrichment analysis. The TF family with *p*-value < 0.01 was considered as the significant over-representation.

To further explore possible functions of these DEGs, *k*-means clustering analysis was performed. DEGs were divided into ten clusters (C1–C10) containing 1480 to 5038 genes in each cluster with distinct expression patterns (Figure 2A; Tables S6 and S7). The first three clusters (C1–C3) contained 3233, 5038 and 2283 DEGs respectively. They showed higher expression levels in 0–6 DAP. These DEGs were significantly (*p*-value < 0.05) enriched for cell division-associated terms, such as DNA replication (GO:0006260), cell cycle (GO:0007049) and microtubule-based process (GO:0007017), indicating frequent cell divisions during the early stage of grain expanding phase (Figure 2B). Besides, genes related to cell differentiation (GO:0030154) and cell growth (GO:0016049) were also enriched in clusters C1–C2 and C2–C3, respectively.

Despite the reversed expression patterns of clusters C2–C3 and C4–C5 (C4, 2915 genes, C5, 2178 genes), they were both involved in embryo development (GO:0009790), consistent with embryo developing events that occurred during this phase of grain development. Moreover, cluster C4 contained genes with largely increased expression in 8–10 DAP, showing significant enrichment for the nutrient reservoir (GO:0045735), glycogen (starch) synthase activity (GO:0004373), and lipid droplet (GO:0005811), inferring the initiating of grain filling. Clusters C6 and C7 contained 2312 and 2437 genes, respectively, displaying preferentially expression in 2–4 DAP and 4–6 DAP. These genes were involved in photosynthesis (GO:0015979) as well as carbohydrate metabolic process (GO:0005975) which may provide substances and energies for the intense cellular activities at these time points. Notably, a significant enrichment for the cellulose biosynthetic process (GO:0030244) in cluster C6 was observed, probably relating to cell wall formation activity during endosperm cellularization. The remaining clusters (clusters C8–C10 with 2627, 1480, and 2389 genes) were expressed highly for specific time points (0, 2, and 6 DAP, respectively), with genes over-represented for other functional classes, such as response to the hormone, defense response, and cell wall organization or biogenesis.

### 3.3. Expression Profiles of Key Genes Involved in Cell Division and Starch Biosynthesis

To investigate the potential molecular mechanism in grain expanding phase, we further studied genes in clusters (C1–C3) that were enriched in cell cycle functions. A total of 108, 164, and 54 cell cycle related genes were detected in clusters C1–C3, respectively. Among them, we found one B-type cyclin-dependent kinase (CDK) *CDKB1-2*, three A-type cyclin *CYCA3-4*, and five D-type cyclin (three *CYCD5-1* and two *CYCD6-1*) in cluster C1 (Figure S4; Table S8), of which *CDKB1-2* and *CYCA3-4* are known to function in G2/M transitions and the D-type cyclin have functions in G1/S transition [28,29].

Compared with cluster C1, C2 contained three *CDKB2-2*, four *CYCA1-1*, five *CYCA2-4*, and five *CYCB1-2* homologs, all of which were annotated to have functions in G2/M transitions. Notably, the orthologs of *CYCB1-2* in rice were known to be critical for endosperm formation via the regulation of mitotic division [30]. In cluster C3, one *CDKA1* involving in G1/S transition, two *CDKB1-2*, three *CYCA3-4* and two *CKS1* (cyclin-dependent kinase-subunit 1) were detected. The ortholog of *CKS1* in Arabidopsis plays an essential role in the regulation of the cell cycle that affects plant growth rate [31]. These results suggest that the cell division precedes the subsequent grain filling.

It is known that the grain filling phase initiates when the endosperm cellularization is finished [32]. Given cluster C4 contained genes enriched in glycogen (starch) synthase activity (Figure 2B) which indicates the initiation of early grain-filling, we chose C4 to investigate the transcription of genes involved in starch and sucrose metabolism in particular. The starch synthesis starts with the production of ADPglucose by AGPase (ADP-glucose pyrophosphorylase) enzyme which is often regarded as the “committed step” of starch synthesis [33]. We found that two *AGPL1* genes encoding large subunits of AGPase and three *AGPS1* genes encoding small subunits expressed highly in 8–10 DAP (Figure S5; Table S9). Besides, genes encoding starch synthase (*SSSIIIa*, 3), granule-bound starch synthase (*GBSSI*, 3) and starch branching enzymes (*SBE*, 8), and debranching enzymes (*ISA1*, 3) were identified in cluster C4, confirming their functions in grain filling.

### 3.4. Differentially Expressed Transcription Factors

Transcription factors (TFs) have crucial roles in controlling plant growth, development and phase changes by regulating gene expression [34]. Of the 5718 TFs identified using the PlantTFDB database [26], 1468 (25.67%) from 50 different families were present during the grain expanding phase, accounting for 5.46% of the total DEGs (Table S10). Among them, *MYB* family members were the most prominent (120), followed by *NAC* (111), *ERF* (91), and *bHLH* (90) (Figure S6). MYB proteins are key factors in regulatory networks controlling development and metabolism under biotic and abiotic stress [35]. The number of TF genes with peak expression levels at 0–6 DAP was more abundant (1188) than that (280) at later time points (8 and 10 DAP) when the grain-filling began (Table S11). Forty-seven (47) out of 50 families had more members in 0–6 DAP, including *ARF* (auxin response factor), *AP2*, *B3*, *GRAS*, and *GRF* (growth-regulating factor) with important roles in plant growth regulation. For example, 31 *ARF* family members (96.77%) were highly expressed in 0–6 DAP, only one (3.23%) was expressed at 8–10 DAP. Whereas, the TF families related to a defense response and storage accumulations, such as *HSF* (heat shock factor) and *NF-YA* (nuclear transcription factor Y subunit alpha) tend to be more abundant in 8–10 DAP.

We further examined the TF numbers in different clusters and found 28 TF families were significantly enriched (*p* < 0.01) in clusters except for C2 and C3 using Fisher’s exact test (Figure 2C). Cluster C5 had the highest TF number (12) and C10 had the lowest (1). Several significantly enriched TF families were identified in the same cluster. For example, CK-responsive B-type *ARR* (Arabidopsis response regulator) genes were only enriched in clusters C6 (30.43%) and C7 (47.83%) with high expressions in 2–6 DAP when the endosperm underwent intense nuclear divisions as observed in Arabidopsis [36]. Moreover, the *HSF* and *NAC* families were significantly enriched in cluster C4. The temporal-specific expression patterns of TFs suggest their distinct roles in regulating wheat grain development and may be good candidates for further investigation.

### 3.5. Dynamic Hormone Metabolism during the Wheat Grain Expanding Phase

**Figure 3:**
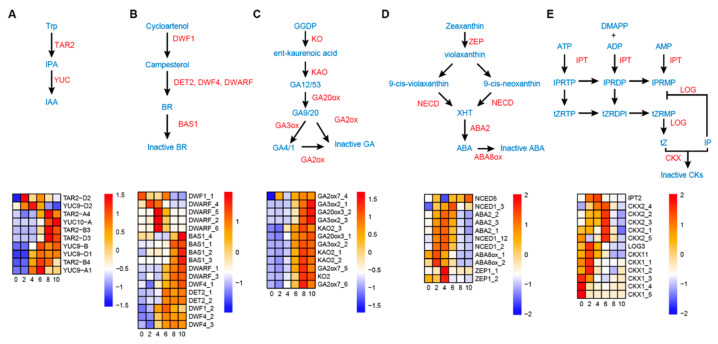
Differential expression patterns of plant hormone metabolism related genes. Diagram of hormone metabolism pathways (upper) and heat maps (lower) of the expression of genes related to the synthesis of auxin (**A**), brassinosteroids (**B**), gibberellic acid (**C**), abscisic acid (**D**), and cytokinin (**E**) across six time points (0, 2, 4, 6, 8, and 10 DAP). The intermediates and enzymes involved in the biosynthesis of various hormones are indicated in blue and red, respectively. The gene expression was displayed based on scaled FPKM values.

Since hormones are the predominant biochemical basis for grain morphogenesis [7], we then analyzed the expression patterns of genes related to IAA, GA, BR, ABA, and CK metabolism in the wheat grain expanding phase. For auxin, genes encoding key biosynthesis enzymes including five *TAR2* (tryptophan aminotransferase related 2), five *YUC* (YUCCA flavin-containing monooxygenase, four *YUC9*, and one *YUC10* were identified most of which showed gradually increased expression levels along the grain development (Figure 3A; Table S12). In comparison, auxin transporters, such as homologs for auxin efflux transporter (*PIN*, 7 genes), auxin influx carrier (*AUX*, 3), and ATP binding cassette B (*ABCB*, 8) had reversed expression patterns, i.e., decreasing over the time of grain development (Figure S7). For BR, multiple genes encoding key biosynthesis enzymes, such as *DWARF1* (*DWF1*, two homologs), *DET2* (deetiolated2, 2), *DWF4* (3), and *DWARF* (6) were found (Figure 3B; Table S12). We observed that the expression profiles of the *DWARF* genes displayed one of two tendencies: high expression at 4 DAP or a gradual increase over the grain expanding phase. *DWARF_4*, *DWARF_5*, and *DWARF_6* manifested the former pattern, while *DWARF_1* and *DWARF_3* showed the latter one. *DWF1* genes also showed two expression patterns with DWF1_1 and DWF1_2 expressing highly in 0–6 DAP and 4–10 DAP, respectively. Other biosynthesis enzymes (two *DET2* and three *DWF4*) and catabolic enzymes (four *BAS1* (PHYB ACTIVATION-TAGGED SUPRESSOR1)) were increased in expression during grain development. For GA metabolism pathways, homologs of nearly all genes for biosynthesis were differentially expressed including one *KO1* (ent-kaurene oxidase), three *KAO2* (ent-kaurenoic acid oxidase), two *GA20ox3* (gibberellin 20-oxidase), three *GA3ox2* (gibberellin 3β-hydroxylase), and three *GA2ox7* (Figure 3C; Table S12). In the ABA biosynthesis pathway, we found homologs to *ZEP1* (zeaxanthin epoxidase1, 2), *NCED1* (9-cis-epoxycarotenoid dioxygenase 1, 3), *NCED5* (1), *ABA2* (ABA DEFICIENT2, 3), and *ABA8ox* (ABA 8′-hydroxylase, 2) genes that consistently highly expressed before 8 DAP with the maximum expression level at 4 DAP (Figure 3D; Table S12). For CK metabolism pathways, 13 cytokinin metabolism related genes, including one homolog of *LONELY GUY* (*LOG*) in Arabidopsis, one isopentenyl transferase (IPT) gene, and 11 putative cytokinin oxidase/dehydrogenase (*CKX*) genes, were identified among the DEGs (Figure 3E; Table S12). The *LOG* and *IPT* exhibited peak expressions in 2–4 DAP, while the expressions of most *CKX* genes remained high before 8 DAP.

### 3.6. Homoeolog Expression Bias during Wheat Grain Development

As a polyploid plant, quantitative variations for many agronomic traits in wheat are determined by multiple sets of homoeologs from the three subgenomes [37]. To explore expression patterns across homoeologs, we focused on 14,284 sets of triads with all three homoeologs available (Table S1). Among them, 6736 (47.16%) had at least one homoeolog differentially expressed and were assigned to any one of the ten co-expression clusters. We found only 1589 (23.59%) of triads with all three homoeologs expressing in the same cluster and 2084 (30.84%) had two homoeologs expressing in the same cluster, demonstrating expression divergence among homoeologs during the wheat grain development.

**Figure 4:**
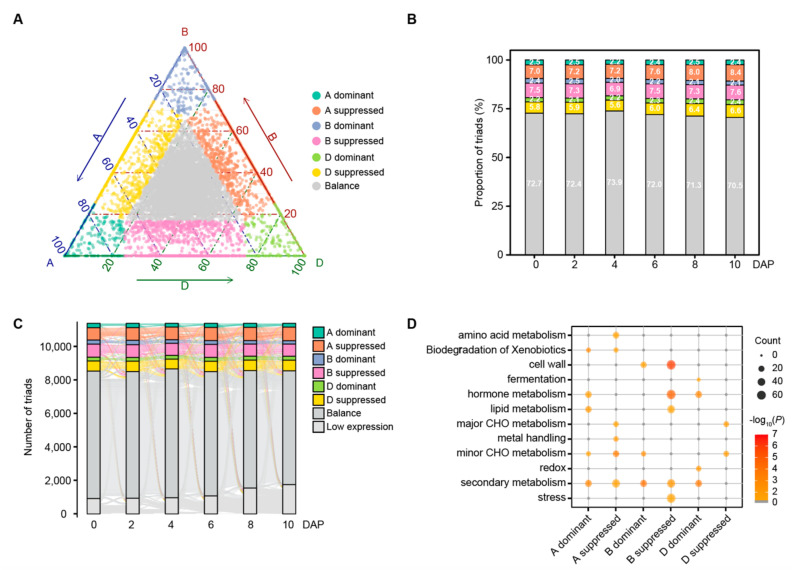
Homoeolog expression bias during the wheat grain expanding phase. (**A**) Ternary plot showing relative expression abundance of triads in 0 DAP as an illustration. Each point represents a triad with an A, B, and D coordinate representing the relative contribution of each homoeolog to the overall triad expression. Triads in vertices correspond to single-subgenome–dominant categories (A dominant, B dominant, and D dominant: expression of one homoeolog beyond those of the others), whereas triads close to edges and between vertices correspond to suppressed categories (A suppressed, B suppressed, and D suppressed: expression of one homoeolog below those of the others). Balanced triads (Balance: similar expressions across three homoeologs) are shown in gray. The dotted lines represent grid lines connecting the labels of the three axes. (**B**) The proportion of triads in each category of homoeolog expression bias at six time points. (**C**) Sankey diagram showing patterns of transition for triads belonging to each category of homoeolog expression bias at different time points. The triad with the sum FPKM values of three homoeolog < 1.0 was assigned as the low expression category. (**D**) Enrichment of metabolism functions among combined triads with at least one differentially expressed homoeologous gene in each dominant and suppressed category across early grain development. A pathway with *p*-value < 0.05 (Fisher’s exact test) was considered significantly enriched.

We then analyzed homoeolog expression bias in all the detected triads. Based on the previously established classification criteria [15], triads (11,398) with the sum FPKM value > 1 were divided into seven categories (See methods) (Figure 4A; Table S13). Most of the triads (72.13% on average) have balanced expression patterns among three homoeologs at six time points. Triads with one-homoeolog suppressed were less frequent (20.97% on average), whereas triads with single-homoeolog dominant were least common (6.90% on average; Figure 4B). Moreover, we also found that 71.82% of balanced triads remained balanced at each of the six time points, whereas dominant and suppressed triads tended to be more variable across the course of grain development, occupying 28.83% and 22.25% of triads studied respectively (Figure 4C).

To investigate the functions of triads with unbalanced homoeolog expression patterns, we firstly obtained 3657 triads with at least one homoeolog differentially expressed (Table S13). Mapman annotation and enrichment analysis showed that triads in different categories were significantly enriched for metabolic pathways including major and minor CHO (carbohydrate) metabolism, secondary metabolism, lipid metabolism, hormone metabolism and cell wall (Figure 4D), suggesting their functions in early grain development. Interestingly, triads in A-homoeolog suppressed or D-homoeolog suppressed categories were enriched in the major CHO metabolism pathway including sugar and starch metabolism (Table S14). Twelve triads were identified for sugar metabolism including *FBP1* (fructose-1,6-bisphosphatase 1) behaving as D-homoeolog suppressed at six time points and so was, *HXK3* (hexokinase *3*) at 6–10 DAP, whereas *CIN2* (cell wall invertase 2) behaved as A-homoeolog suppressed at 0–4 DAP (Figure S8). For starch metabolism, the expression of one *SSSII* gene was found to be mainly contributed by A and B homoeologs, while the second *SBE* gene was dominantly contributed by B and D homoeologs (Figure S8). On the other hand, hormone-related triads (91) also accounted for a significant proportion in categories of A dominant, B suppressed, and D dominant, suggesting the dominant influence of A and D subgenome for hormone functions in wheat grain development. Among these triads, enzymes for hormone metabolism, such as *YUC10*, *AOS2* (allene oxide synthase 2), and *CYP90D2* (cytochrome P450 90D2) displayed A dominant expression patterns at 6–10 DAP, and others, such as *CKX1_2* and *ZEP1*, exhibited B suppressed expression patterns at 2 DAP, a time point when the free-nuclear division occurred in the endosperm. Notably, the *YUC10* rice ortholog *OsYUC11* has been shown to be a key contributor to auxin biosynthesis in endosperm and is essential for grain filling [38]. In addition, homologs of *AOS4* and auxin efflux transporters (*PIN1* and *PIN7*) showed B suppressed or D dominant expression patterns at 0–6 DAP. Other hormone related genes, such as homologs of the gibberellin receptor *GID1C* and auxin responsive genes (*GH3-4* and *SAUR37*) also displayed unbalanced expression patterns at some time points (Table S15; Figure S9). Understanding the homoeolog expression patterns will help inform strategies to improve crops by targeting and manipulating individual or multiple homoeologs to quantitatively modulate grain development.

## 4. Discussion

The early development of wheat grain is critical for final grain yield. Compared with previous studies on wheat early grain development based on microarray technology [5,6], RNA-seq technology has obvious advantages for transcriptome dynamics analysis. Our comprehensive analysis of time-series transcriptomes during the wheat grain expanding phase revealed a large number of DEGs over the developing course, indicating dynamic and complex gene regulation, consistent with previous observations in the wheat endosperm [1].

Cereal grain development often begins with double fertilization that generates diploid embryos and triploid endosperms. During early developmental stages, the embryo and endosperm grow rapidly through cell division and growth in a coordinated manner that is affected by the maternal tissues likely the pericarp [39]. We showed here high expression levels of cell cycle and starch biosynthesis related genes, specifically in 0–6 DAP and the following 8–10 DAP, respectively, suggesting that these genes contributed to grain expanding and the initiation of grain filling in wheat. Notably, several key regulators of the mitotic cycle process, such as the *CYCB1-2* [30] and *CKS1* [31], as well as the rate-limiting enzymes for starch biosynthesis (*AGPL1* and *AGPS1*) [33] were observed and could be potential targets for future molecular verification and breeding application.

Transcription factors are major factors for signal transduction and gene expression regulation and play important roles in plant growth, development, and stress responses. In this study, we found several TF families that were related to cell proliferation and growth, such as *ARF*, B-type *ARR* and *GRF* expressed that were highly expressed in 0–6 DAP. Such expression patterns are consistent with the developmental changes including the formation of syncytium and the fast pericarp growth during the early grain development. Interestingly, as shown in Figure S10, *TaARF25*, which is expressed highly in 0- and 4-DAP outer pericarps, has been shown to positively regulate grain size and weight in our previous study [40]. Moreover, the regulations of the early grain-filling process may be regulated by other TF families, such as *NF-YA* and *NAC* with high expression levels in 8–10 DAP, consistent with previous reports [41,42,43]. We identified a known wheat grain quality regulator of starch- and seed storage protein-related genes, *TaNAC019* [42], which showed a rapid increase in expression level from 8 DAP (Figure S11). Plant *HSF* genes are expressed not only in response to stress but also during various developmental programs, including pollen development [44] and seed maturation [45]. The gradually increasing expression of *HSF* genes from 0–10 DAP may suggest their roles in early grain filling under adverse conditions.

Hormonal regulation of grain development and their dynamic changes during grain development is widely studied in plants, such as Arabidopsis, maize and rice [7,46,47]. We observed distinct expression patterns of numerous genes for the metabolism of various hormones. The biosynthesis gene for ABA and CK tend to be expressed in 0–6 DAP, those for auxin and GA in 8–10 DAP, while those for BR were expressed across the developmental period. The sequential biosynthesis of different hormones may suggest their distinct functions on wheat grain development. In rice, CK and BR, for example, may be involved in regulating cell division at both the free nuclear (2 DAP) and cellularization (3 DAP) stages in endosperm as shown in early grain development [46]. In wheat, one BR biosynthesis gene (*DWARF*) and nearly all ABA biosynthesis genes were present at 2–4 DAP in addition to CK biosynthesis genes, suggesting a largely conserved mode of action for these key regulatory genes in the two grass species. Other BR biosynthesis (*DWARF*, *DWF4*, *DET2*, and *DWF1*) and catabolic genes (*BAS1*) tend to express in the later stages to regulate endosperm cell expansion, a well-known effect of BR on grain size development [12,46].

Auxin is a key regulator of grain development, orchestrating cell division, elongation, differentiation, and embryonic development [7]. It is known that *de novo* synthesis of auxin occurs during grain development and is associated with embryonic development and endosperm starch biosynthesis [38,48,49]. We found that most *TAR* and *YUC* genes were expressed highly from 8 DAP when the grain filling started, except for *TAR2-D2* and *YUC9-D2* which showed specific expressions in 2–6 DAP. In particular, the *TAR2-A4*, *TAR2-B3*, *TAR2-D3*, and *YUC10-A* were assigned in cluster C4 and co-expressed with genes for starch biosynthesis enzymes (Figure S6), suggesting a possible relationship between these two processes in grain filling. In addition to genes for *de novo* biosynthesis, auxin transport proteins were responsible for the differential auxin distribution to shape organs through the mediation of polar auxin transport [50]. We found differentially expressed transporter genes including *PIN1/2/7*, *AUX1* and *ABCB1/19* genes, which have been shown to participate in multiple developmental processes including embryogenesis, endosperm initiation, and pericarp extension [11,51,52], highly expressed in 0–6 DAP, suggesting their potential roles on the embryogenesis, endosperm initiation and pericarp growth. Converse to ABA, GA has antagonistic roles in the regulation of signaling molecules and gene expression, contributing to concentrations of calcium, α-amylase, MAP kinase, and PH [53]. Nearly all the identified GA metabolism genes, such as those for biosynthesis (*KO*, *KAO*, *GA20ox*, and *GA3ox*) and catabolism (*GA2ox*) expressed with an increasing level along the grain development, particularly at 8–10 DAP, suggesting their potential effect on starch degradation so as to affect grain quality. Taken together, hormones likely regulate multiple biological processes in early grain development and the comprehensive expression profiling should provide useful information for further understanding of hormone regulatory mechanisms in wheat.

Investigating homoeolog expression bias may help pinpoint actively expressed genes as targets for genetic improvement. The bias of expression among homoeologs contributed differentially to various morphological and agronomical traits, such as the production of rRNA and storage proteins, and in interaction with pathogens [1,54]. We found about 30% of homoeolog triads showing unbalanced expression patterns, similar to a recent report in wheat [15]. Triads with unbalanced expression patterns appear to be variable, with 28.83% being A-, B-, and D-dominant and 22.25% as A-, B-, and D-suppressed, showing a strong transcriptional reprogramming at the grain expanding phase. Functional enrichment analysis showed that these imbalance triads were enriched in multiple specific metabolic pathways that may be critical for wheat grain development. For instance, among triads associated with sugar and starch metabolism, expressions of A or D homoeologs tended to be suppressed, indicating unbalanced contributions from B homoeologs on these important pathways, while A and D homoeologs contributed to hormone metabolism triads. One example was *AOS2/4*, a key biosynthesis enzyme gene for jasmonate whose expression was contributed to by A- and D-homoeologs. Previous work also showed biased contributions from A and D sub-genomes on the expression of a *lipoxygenase* gene that catalyzes the first step in the jasmonate biosynthesis pathway under pathogen-inoculated conditions [17]. Differences in expression levels among the three homoeologs may be associated with various regulation elements, such as cis- and trans-regulatory elements, DNA methylation and histone modifications [15]. These underlying mechanisms for unbalanced homoeolog expression need further investigation.

## 5. Conclusions

Systematic investigation of transcriptome dynamics during wheat grain expanding phase (0–10 DAP) identified genes with specific expression patterns that may reflect their important roles in cell division and early grain-filling during wheat early grain development. The work elucidates the potential regulatory mechanisms of the cell cycle, starch biosynthesis and hormone metabolism, and homoeolog expression bias in this process and may provide candidate genes for further functional characterization. The knowledge may serve as a valuable resource to develop molecular markers for wheat yield improvement.

## Data Availability

The data presented in this study are openly available in the Sequence Read Archive of the National Center for Biotechnology Information (NCBI) under BioProject PRJNA780107.

## Supplementary Materials

The following supporting information can be downloaded at: www.mdpi.com/article/10.3390/biology11020281/s1, Figure S1: Selection of an optimal number of clusters based on Silhouette scores for *k*-means clustering approach. The dashed line in the plot indicates the best cluster number *k*; Figure S2: Number of DEGs (differentially expressed genes) between adjacent time points (0 DAP vs. 2 DAP; 2 DAP vs. 4 DAP; 4 DAP vs. 6 DAP; 6 DAP vs. 8 DAP; 8 DAP vs. 10 DAP) during wheat early grain development; Figure S3: DEG distribution along the three wheat subgenomes. DEG density was counted with 1-Mb windows; Figure S4: Heatmap showing the expression profiles of cell cycle related genes in co-expression clusters C1 (A), C2 (B), and C3 (C). The gene expression was displayed based on scaled FPKM values; Figure S5: Diagram (A) displaying the starch biosynthesis pathway and heatmap (B) showing the expression profiles of starch biosynthesis related genes in co-expression cluster C4. The gene expression was displayed based on scaled FPKM values; Figure S6: Number of transcription factors (TFs) differentially expressed between adjacent time points (0 DAP vs. 2 DAP; 2 DAP vs. 4 DAP; 4 DAP vs. 6 DAP; 6 DAP vs. 8 DAP; 8 DAP vs. 10 DAP) during wheat early grain development; Figure S7: Heatmap showing expression profiles of auxin transporter genes differentially expressed during six time points. Figure S8: Bar plot of homeologous genes related to sugar metabolism and starch biosynthesis with unbalanced expression patterns. Bars of different colors represent different homoeolog of the A, B, and D subgenomes. Tri-angles with different colors represent different categories of unbalanced expression patterns; Figure S9: Bar plot of homoeologous genes related to hormone metabolism and signaling with unbalanced expression patterns. Bars with different colors represent different homoeologs from A, B, and D subgenomes. Tri-angles with different colors represent different categories of unbalanced expression patterns; Figure S10: Expression levels of *TaARF25* homoeologs from A, B, and D genomes respectively during six time points; Figure S11: Expression levels of *TaNAC109* homoeologs from A, B, and D genomes respectively in co-expression cluster C4 at six time points; Table S1: List of triads identified based on protein sequences; Table S2: Statistics of sequencing data and alignment; Table S3: Spearman Correlation Coefficients among three biological replicates based on the FPKM values of expressed genes (FPKM > 1); TableS4: List of DEGs (differentially expressed genes) between adjacent time points; Table S5: Significant GO enrichment for DEGs (differentially expressed genes) between adjacent time points; Table S6: Genes assigned to ten co-expression clusters by *k*-means clustering approach; Table S7: Numbers of genes assigned to ten co-expression clusters by *k*-means clustering approach; Table S8: List of DEGs (differentially expressed genes) associated with cell cycle in co-expression clusters C1–C3; Table S9: List of DEGs (differentially expressed genes) associated with starch biosynthesis in co-expression cluster C4; Table S10: Genome-wide transcription factors (TFs) identification based on protein sequences using PlantTFDB database; Table S11: Numbers of transcription factors (TFs) with peak expression in pre-grain-filling and early grain-filling stages; Table S12: List of DEGs (differentially expressed genes) associated with hormone metabolism; Table S13: Triads in the seven categories of homoeolog expression patterns in each of six time points; Table S14: Triads related to major CHO (carbohydrate) metabolism with unbalanced homoeolog expression patterns; Table S15: Triads related to hormone metabolism with unbalanced homoeolog expression patterns.

## References

[1] Pfeifer M., Kugler K.G., Sandve S.R., Zhan B., Rudi H., Hvidsten T.R., Mayer K.F.X., Olsen O.-A. (2014). International Wheat Genome Sequencing Consortium Genome interplay in the grain transcriptome of hexaploid bread wheat. Science.

[2] Mechin V., Thevenot C., Le Guilloux M., Prioul J.L., Damerval C. (2007). Developmental analysis of maize endosperm proteome suggests a pivotal role for pyruvate orthophosphate dikinase. Plant Physiol..

[3] Yan L., Liu Z., Xu H., Zhang X., Zhao A., Liang F., Xin M., Peng H., Yao Y., Sun Q. (2018). Transcriptome analysis reveals potential mechanisms for different grain size between natural and resynthesized allohexaploid wheats with near-identical AABB genomes. BMC Plant Biol..

[4] Saulnier L., Guillon F., Chateigner-Boutin A.-L. (2012). Cell wall deposition and metabolism in wheat grain. J. Cereal Sci..

[5] Capron D., Mouzeyar S., Boulaflous A., Girousse C., Rustenholz C., Laugier C., Paux E., Bouzidi M.F. (2012). Transcriptional profile analysis of E3 ligase and hormone-related genes expressed during wheat grain development. BMC Plant Biol..

[6] Shewry P.R., Mitchell R.A., Tosi P., Wan Y., Underwood C., Lovegrove A., Freeman J., Toole G.A., Mills E.C., Ward J.L. (2012). An integrated study of grain development of wheat (cv. Hereward). J. Cereal Sci..

[7] Locascio A., Roig-Villanova I., Bernardi J., Varotto S. (2014). Current perspectives on the hormonal control of seed development in Arabidopsis and maize: A focus on auxin. Front. Plant Sci..

[8] Basunia M., Nonhebel H.M. (2019). Hormonal regulation of cereal endosperm development with a focus on rice (Oryza sativa). Funct. Plant Biol..

[9] Rijavec T., Dermastia M. (2010). Cytokinins and their Function in Developing Seeds. Acta Chim. Slov..

[10] Yang J., Zhang J., Huang Z., Wang Z., Zhu Q., Liu L. (2002). Correlation of cytokinin levels in the endosperms and roots with cell number and cell division activity during endosperm development in rice. Ann. Bot..

[11] Friml J., Vieten A., Sauer M., Weijers D., Schwarz H., Hamann T., Offringa R., Jurgens G. (2003). Efflux-dependent auxin gradients establish the apical-basal axis of Arabidopsis. Nature.

[12] Li N., Xu R., Li Y. (2019). Molecular Networks of Seed Size Control in Plants. Annu. Rev. Plant Biol..

[13] Cheng Z.J., Zhao X.Y., Shao X.X., Wang F., Zhou C., Liu Y.G., Zhang Y., Zhang X.S. (2014). Abscisic Acid Regulates Early Seed Development in Arabidopsis by ABI5-Mediated Transcription of SHORT HYPOCOTYL UNDER BLUE1. Plant Cell.

[14] Marcussen T., Sandve S.R., Heier L., Spannagl M., Pfeifer M., Jakobsen K.S., Wulff B.B.H., Steuernagel B., Mayer K.F.X., Olsen O.-A. (2014). Ancient hybridizations among the ancestral genomes of bread wheat. Science.

[15] Ramirez-Gonzalez R.H., Borrill P., Lang D., Harrington S.A., Brinton J., Venturini L., Davey M., Jacobs J., van Ex F., Pasha A. (2018). The transcriptional landscape of polyploid wheat. Science.

[16] Leach L.J., Belfield E.J., Jiang C., Brown C., Mithani A., Harberd N.P. (2014). Patterns of homoeologous gene expression shown by RNA sequencing in hexaploid bread wheat. BMC Genom..

[17] Powell J.J., Fitzgerald T.L., Stiller J., Berkman P.J., Gardiner D.M., Manners J.M., Henry R.J., Kazan K. (2017). The defence-associated transcriptome of hexaploid wheat displays homoeolog expression and induction bias. Plant Biotechnol. J..

[18] Liu Z., Xin M., Qin J., Peng H., Ni Z., Yao Y., Sun Q. (2015). Temporal transcriptome profiling reveals expression partitioning of homeologous genes contributing to heat and drought acclimation in wheat (*Triticum aestivum* L.). BMC Plant Biol..

[19] Chen S., Zhou Y., Chen Y., Gu J. (2018). fastp: An ultra-fast all-in-one FASTQ preprocessor. Bioinformatics.

[20] Dobin A., Davis C.A., Schlesinger F., Drenkow J., Zaleski C., Jha S., Batut P., Chaisson M., Gingeras T.R. (2013). STAR: Ultrafast universal RNA-seq aligner. Bioinformatics.

[21] Trapnell C., Roberts A., Goff L., Pertea G., Kim D., Kelley D.R., Pimentel H., Salzberg S.L., Rinn J.L., Pachter L. (2012). Differential gene and transcript expression analysis of RNA-seq experiments with TopHat and Cufflinks. Nat. Protoc..

[22] Niknafs Y.S., Pandian B., Iyer H.K., Chinnaiyan A.M., Iyer M.K. (2017). TACO produces robust multisample transcriptome assemblies from RNA-seq. Nat. Methods.

[23] Anders S., Pyl P.T., Huber W. (2015). HTSeq--a Python framework to work with high-throughput sequencing data. Bioinformatics.

[24] Love M.I., Huber W., Anders S. (2014). Moderated estimation of fold change and dispersion for RNA-seq data with DESeq2. Genome Biol..

[25] Yu G., Wang L.G., Han Y., He Q.Y. (2012). clusterProfiler: An R package for comparing biological themes among gene clusters. OMICS.

[26] Zhang H., Jin J., Tang L., Zhao Y., Gu X., Gao G., Luo J. (2011). PlantTFDB 2.0: Update and improvement of the comprehensive plant transcription factor database. Nucleic Acids Res..

[27] Emms D.M., Kelly S. (2019). OrthoFinder: Phylogenetic orthology inference for comparative genomics. Genome Biol..

[28] Dante R.A., Larkins B.A., Sabelli P.A. (2014). Cell cycle control and seed development. Front. Plant Sci..

[29] Inze D., De Veylder L. (2006). Cell cycle regulation in plant development. Annu. Rev. Genet..

[30] Guo J., Wang F., Song J., Sun W., Zhang X.S. (2010). The expression of Orysa;CycB1;1 is essential for endosperm formation and causes embryo enlargement in rice. Planta.

[31] De Veylder L., Beemster G.T., Beeckman T., Inze D. (2001). CKS1At overexpression in Arabidopsis thaliana inhibits growth by reducing meristem size and inhibiting cell-cycle progression. Plant J..

[32] Opanowicz M., Hands P., Betts D., Parker M.L., Toole G.A., Mills E.N., Doonan J.H., Drea S. (2011). Endosperm development in Brachypodium distachyon. J. Exp. Bot..

[33] Martin C., Smith A.M. (1995). Starch biosynthesis. Plant Cell.

[34] Ramachandran S., Hiratsuka K., Chua N.H. (1994). Transcription factors in plant growth and development. Curr. Opin. Genet. Dev..

[35] Dubos C., Stracke R., Grotewold E., Weisshaar B., Martin C., Lepiniec L. (2010). MYB transcription factors in Arabidopsis. Trends Plant Sci..

[36] Day R.C., Herridge R.P., Ambrose B.A., Macknight R.C. (2008). Transcriptome analysis of proliferating Arabidopsis endosperm reveals biological implications for the control of syncytial division, cytokinin signaling, and gene expression regulation. Plant Physiol..

[37] Borrill P., Adamski N., Uauy C. (2015). Genomics as the key to unlocking the polyploid potential of wheat. New Phytol..

[38] Xu X., E Z., Zhang D., Yun Q., Zhou Y., Niu B., Chen C. (2021). OsYUC11-mediated auxin biosynthesis is essential for endosperm development of rice. Plant Physiol..

[39] Olsen O.A. (2004). Nuclear endosperm development in cereals and Arabidopsis thaliana. Plant Cell.

[40] Jia M., Li Y., Wang Z., Tao S., Sun G., Kong X., Wang K., Ye X., Liu S., Geng S. (2021). TaIAA21 represses TaARF25-mediated expression of TaERFs required for grain size and weight development in wheat. Plant J..

[41] Xu J.-J., Zhang X.-F., Xue H.-W. (2016). Rice aleurone layer specific OsNF-YB1 regulates grain filling and endosperm development by interacting with an ERF transcription factor. J. Exp. Bot..

[42] Gao Y., An K., Guo W., Chen Y., Zhang R., Zhang X., Chang S., Rossi V., Jin F., Cao X. (2021). The endosperm-specific transcription factor TaNAC019 regulates glutenin and starch accumulation and its elite allele improves wheat grain quality. Plant Cell.

[43] Zhang Z., Dong J., Ji C., Wu Y., Messing J. (2019). NAC-type transcription factors regulate accumulation of starch and protein in maize seeds. Proc. Natl. Acad. Sci. USA.

[44] Renak D., Gibalova A., Solcova K., Honys D. (2014). A new link between stress response and nucleolar function during pollen development in Arabidopsis mediated by AtREN1 protein. Plant Cell Environ..

[45] Kotak S., Vierling E., Baumlein H., von Koskull-Doring P. (2007). A novel transcriptional cascade regulating expression of heat stress proteins during seed development of Arabidopsis. Plant Cell.

[46] Zhang X., Tong J., Bai A., Liu C., Xiao L., Xue H. (2020). Phytohormone dynamics in developing endosperm influence rice grain shape and quality. J. Integr. Plant Biol..

[47] Sun X., Shantharaj D., Kang X., Ni M. (2010). Transcriptional and hormonal signaling control of Arabidopsis seed development. Curr. Opin. Plant Biol..

[48] Cheng Y., Dai X., Zhao Y. (2007). Auxin synthesized by the YUCCA flavin Monooxygenases is essential for embryogenesis and leaf formation in Arabidopsis. Plant Cell.

[49] McAdam E.L., Meitzel T., Quittenden L.J., Davidson S.E., Dalmais M., Bendahmane A.I., Thompson R., Smith J.J., Nichols D.S., Urquhart S. (2017). Evidence that auxin is required for normal seed size and starch synthesis in pea. New Phytol..

[50] Smith R.S., Bayer E.M. (2009). Auxin transport-feedback models of patterning in plants. Plant Cell Environ..

[51] Ugartechea-Chirino Y., Swarup R., Swarup K., Peret B., Whitworth M., Bennett M., Bougourd S. (2010). The AUX1 LAX family of auxin influx carriers is required for the establishment of embryonic root cell organization in Arabidopsis thaliana. Ann. Bot..

[52] Cao J., Li G., Qu D., Li X., Wang Y. (2020). Into the Seed: Auxin Controls Seed Development and Grain Yield. Int. J. Mol. Sci..

[53] Bethke P.C., Schuurink R., Jones R.L. (1997). Hormonal signalling in cereal aleurone. J. Exp. Bot..

[54] Feldman M., Levy A.A., Fahima T., Korol A. (2012). Genomic asymmetry in allopolyploid plants: Wheat as a model. J. Exp. Bot..

