# Supplementary material for: Transcriptome Analysis of Developing Wheat Grains at Rapid Expanding Phase Reveals Dynamic Gene Expression Patterns
Source: Biology (Basel). 2022 Feb 10;11(2):281. doi: 10.3390/biology11020281 (PMC8869726; doi:10.3390/biology11020281)
Supplement: Supplementary file 1 [file biology-11-00281-s001.zip › biology-1530986-supplementary/Supplementary.Figure.Proof.pdf]

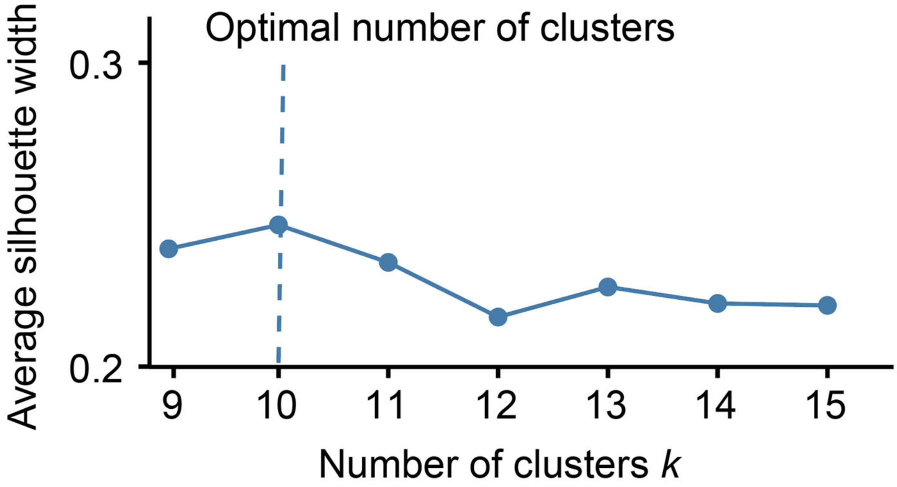

Figure S1. Selection of an optimal number of clusters based on Silhouette scores for *k*-means clustering approach. The dash line in the plot indicates the best cluster number *k*.

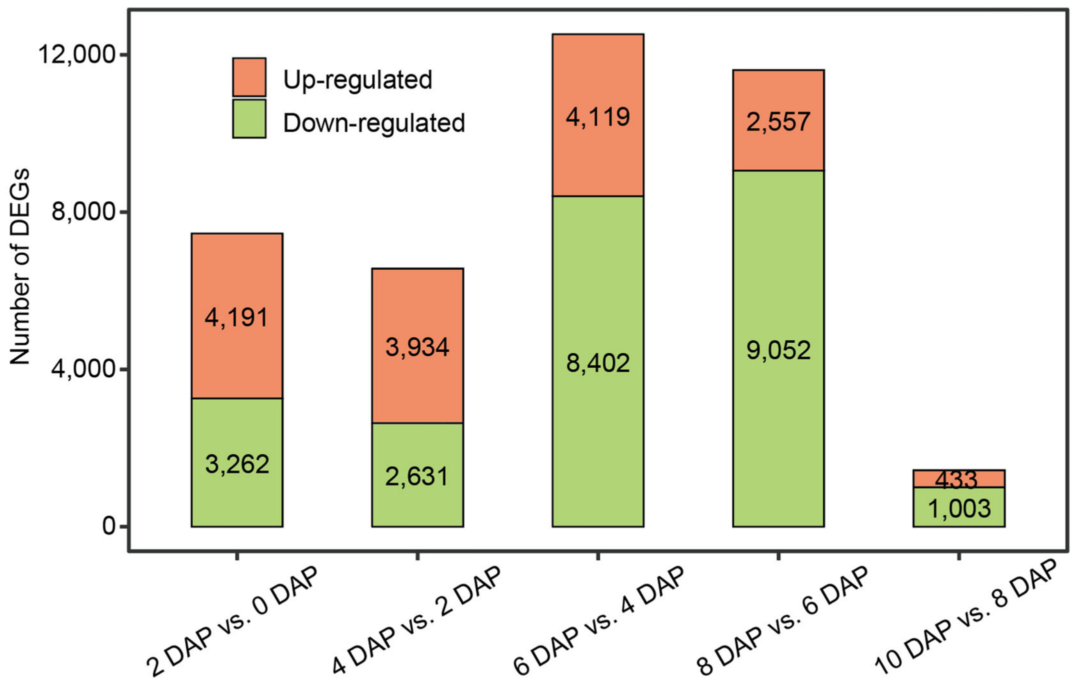

Figure S2. Number of DEGs (differentially expressed genes) between adjacent time points (0 DAP vs. 2 DAP; 2 DAP vs. 4 DAP; 4 DAP vs. 6 DAP; 6 DAP vs. 8 DAP; 8 DAP vs. 10 DAP) during wheat early grain development.

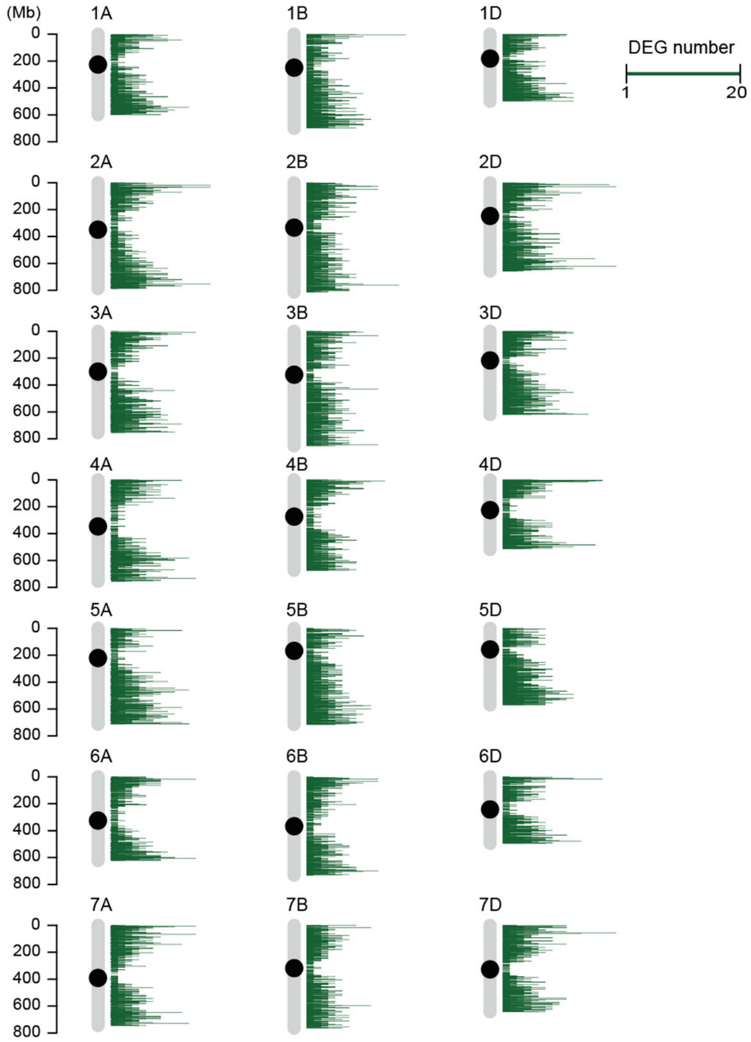

Figure S3. DEG distribution along the three wheat subgenomes. DEG density was counted with 1-Mb win-dows.

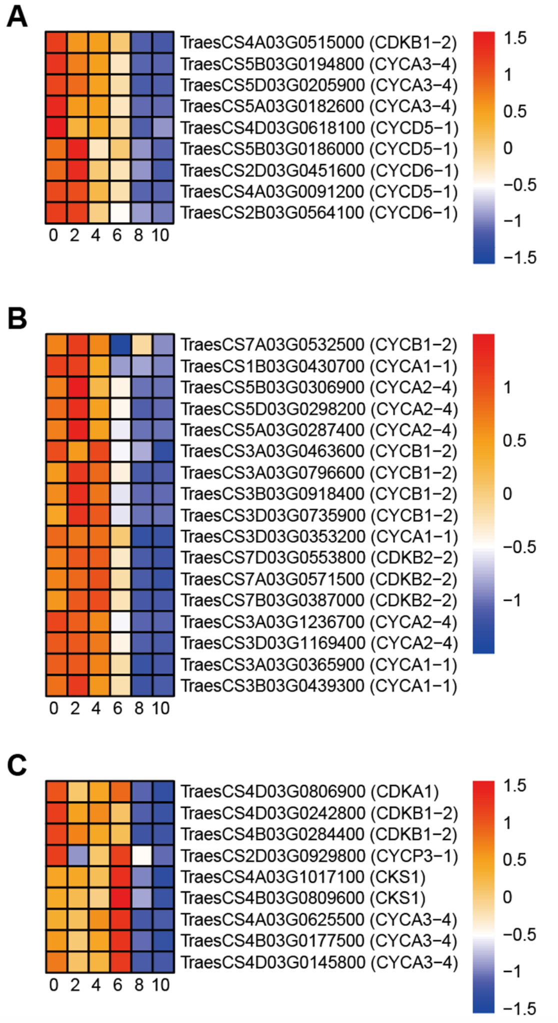

Figure S4. Heatmap showing the expression profiles of cell cycle related genes in co-expression clusters C1 (**A**), C2 (**B**), and C3 (**C**). The gene expression was displayed based on scaled FPKM values.

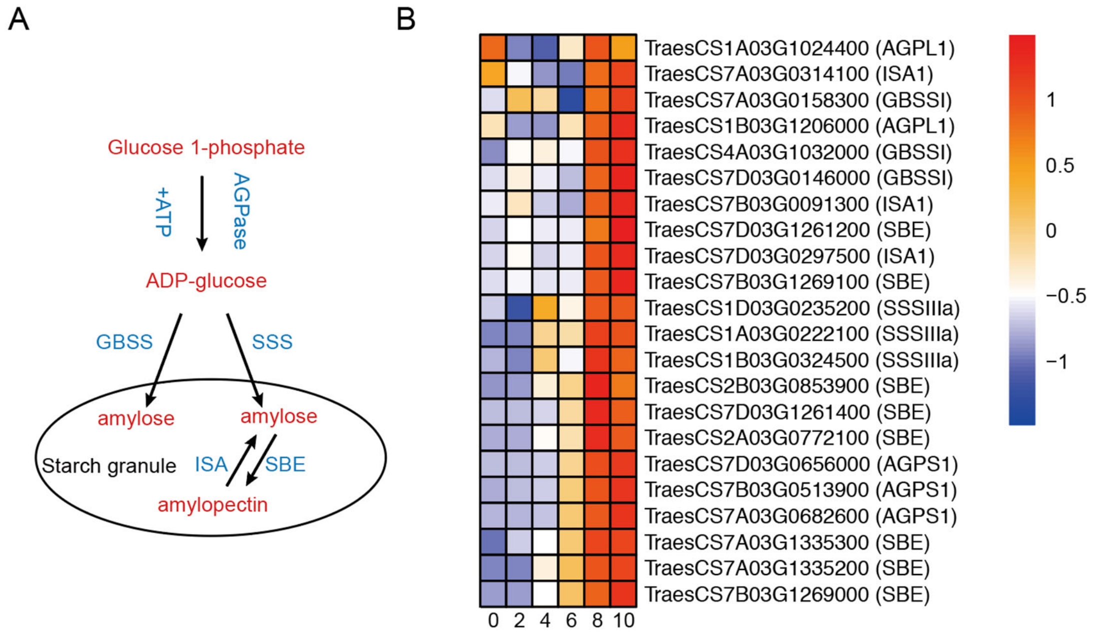

Figure S5. Diagram (**A**) displaying the starch biosynthesis pathway and heatmap (**B**) showing the expression profiles of starch biosynthesis related genes in co-expression cluster C4. The gene expression was displayed based on scaled FPKM values.

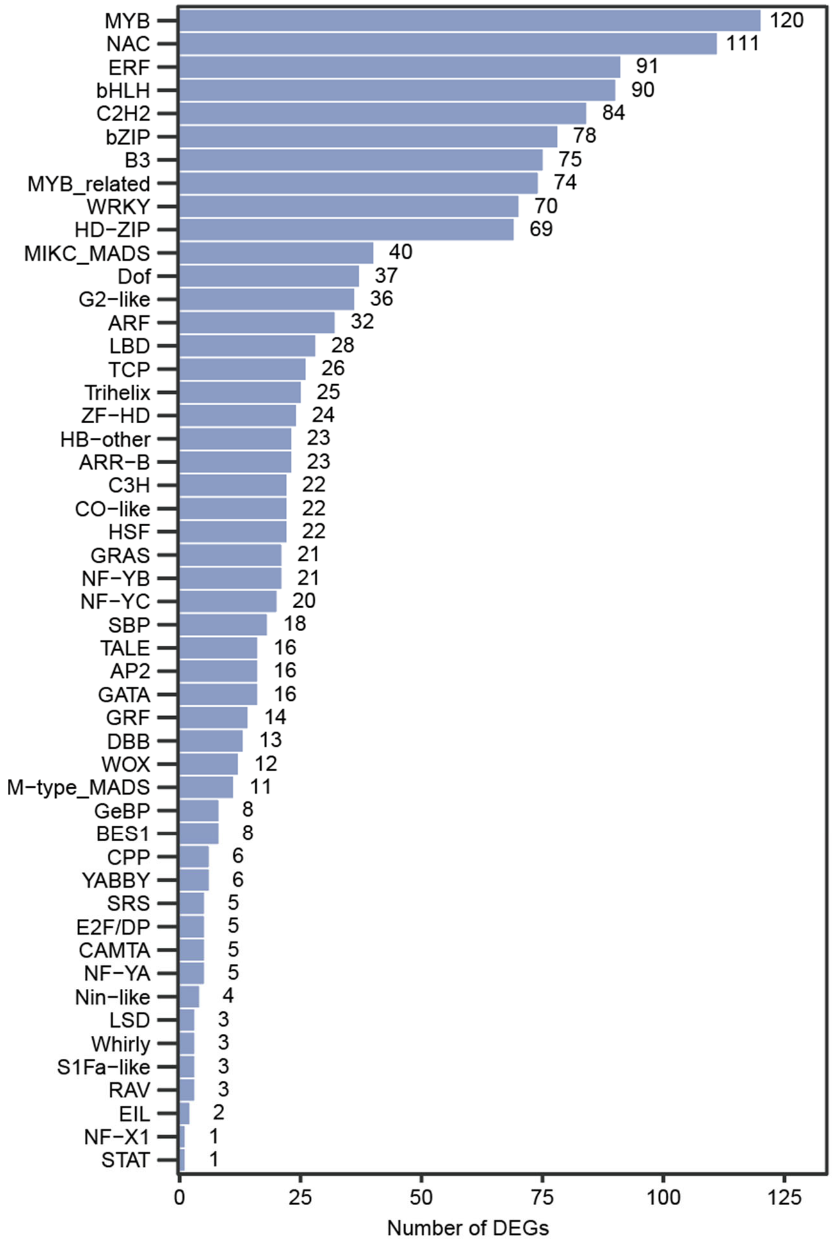

Figure S6. Number of transcription factors (TFs) differentially expressed between adjacent time points (0 DAP vs. 2 DAP; 2 DAP vs. 4 DAP; 4 DAP vs. 6 DAP; 6 DAP vs. 8 DAP; 8 DAP vs. 10 DAP) during wheat early grain development.

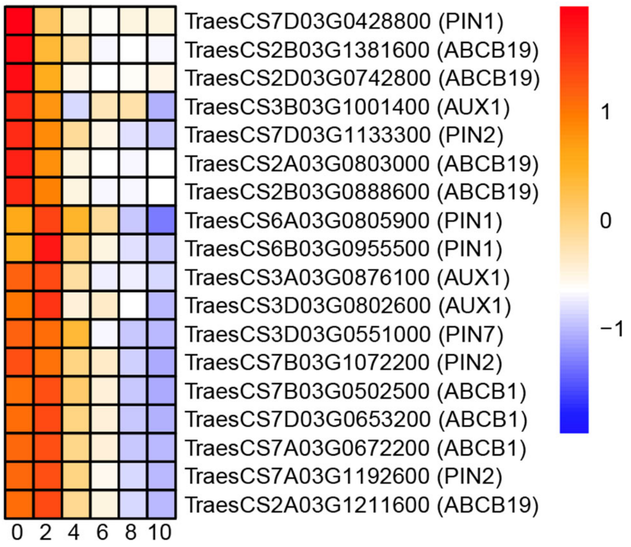

Figure S7. Heatmap showing expression profiles of auxin transporter genes differentially expressed during six time points.

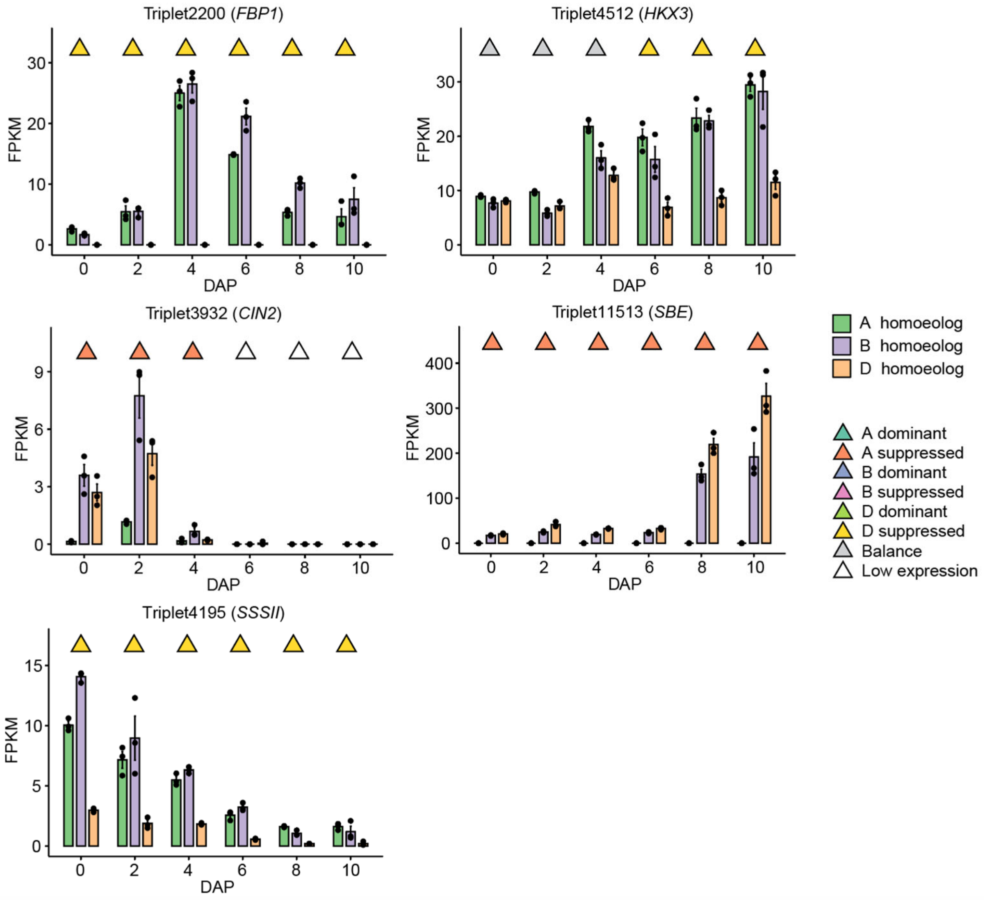

Figure S8. Bar plot of homeologous genes related to sugar metabolism and starch biosynthesis with unbalanced expression patterns. Bars of different colors represent different homoeolog of the A, B, and D subgenomes. Tri-angles with different colors represent different categories of unbalanced expression patterns.

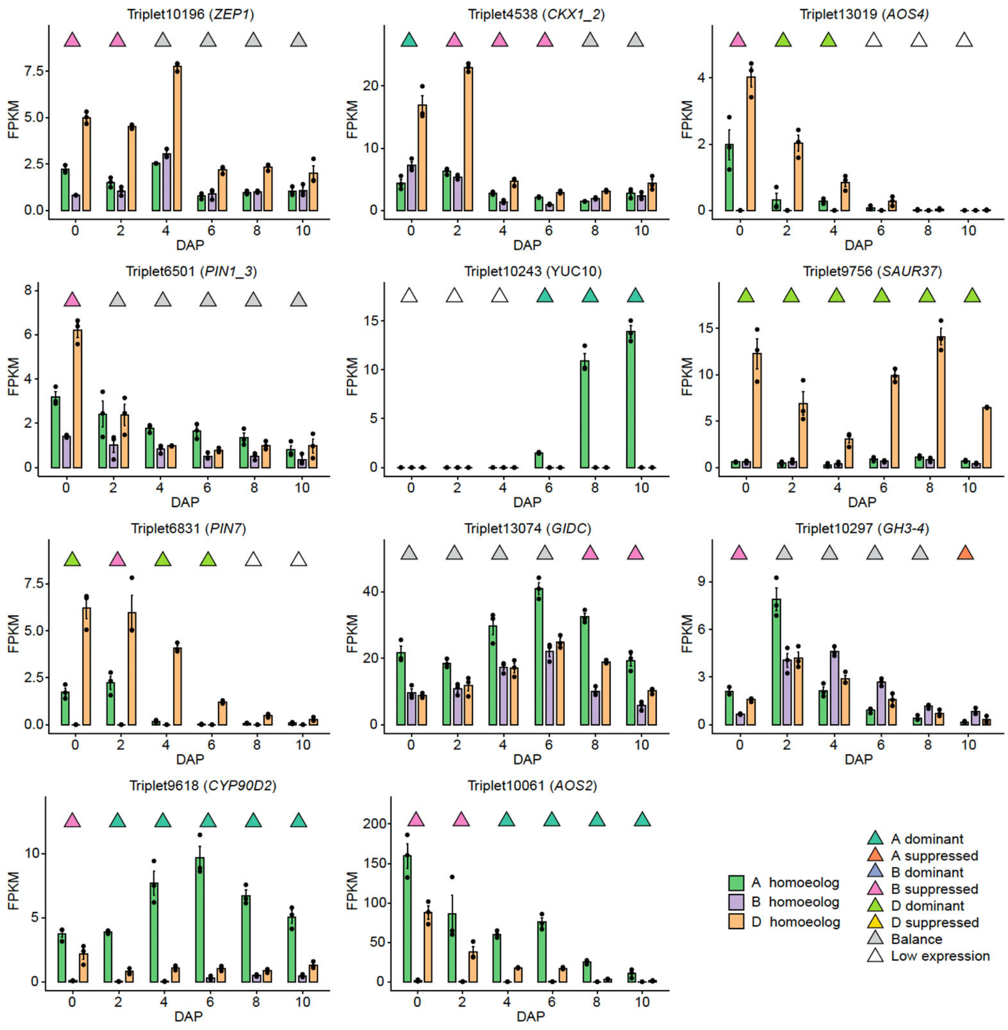

Figure S9. Bar plot of homoeologous genes related to hormone metabolism and signaling with unbalanced expression patterns. Bars with different colors represent different homoeologs from A, B, and D subgenomes. Tri-angles with different colors represent different categories of unbalanced expression patterns.

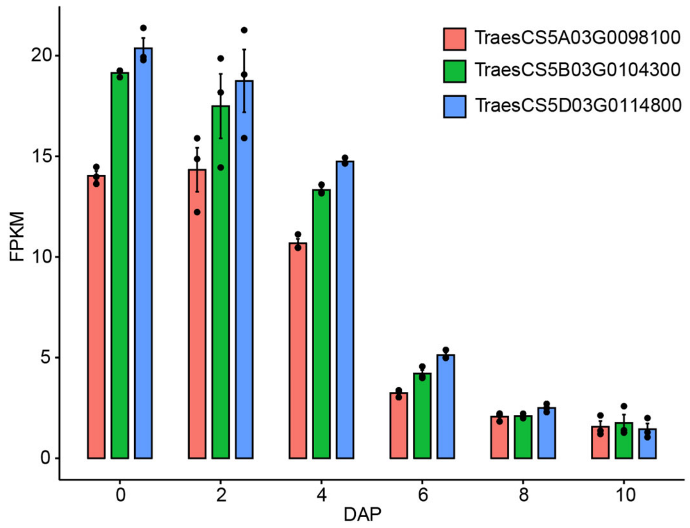

Figure S10. Expression levels of *TaARF25* homoeologs from A, B, and D genomes respectively during six time points.

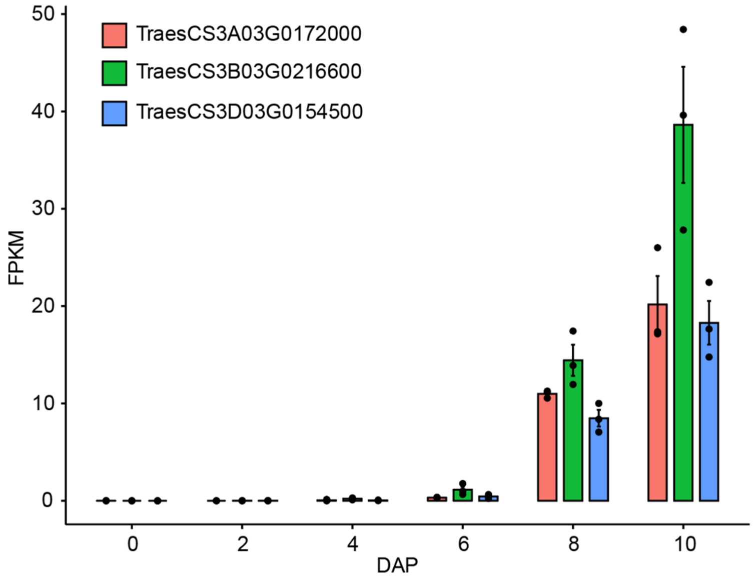

Figure S11. Expression levels of *TaNAC109* homoeologs from A, B, and D genomes respectively in co-expression cluster C4 at six time points.
